# Adult Migraine Hospital Admission Trends in Finland: A Nationwide Registry Study

**DOI:** 10.3390/jcm9020320

**Published:** 2020-01-23

**Authors:** Jussi O.T. Sipilä, Jori O. Ruuskanen, Päivi Rautava, Ville Kytö

**Affiliations:** 1 Siun sote, North Karelia Central Hospital, Department of Neurology, 80210 Joensuu, Finland; 2 Division of Clinical Neurosciences, Department of Neurology, Turku University Hospital, 20521 Turku, Finland; jori.ruuskanen@tyks.fi; 3 Clinical Neurosciences, University of Turku, 20520 Turku, Finland; 4 Department of Public Health, University of Turku, 20520 Turku, Finland; paivi.rautava@tyks.fi; 5 Turku Clinical Research Centre, Turku University Hospital, 20521 Turku, Finland; 6 Heart Center, Turku University Hospital, 20521 Turku, Finland; vijoky@utu.fi; 7 Research Center of Applied and Preventive Cardiovascular Medicine, University of Turku, 20520 Turku, Finland

**Keywords:** cardiovascular risk, epidemiology, headache, migraine

## Abstract

Population-level data on migraine hospital admission trends are unavailable. Changes in stroke care may have influenced these, since migraine is one of the most common stroke mimics. In this study, all hospital admissions on neurological, internal medicine, and pediatric wards in Finland with migraine as the primary diagnosis for persons at least 16 years of age in 2004–2014 were studied, resulting in an analysis of 6195 individuals with 7764 migraine discharges. The number of discharges increased by 4.2% annually (*p* = 0.000084), with no change in age of the admitted patients. Comorbidity burden was low but increased during the study period (*p* < 0.0001). The frequency of common vascular risk factors as comorbidities increased by 11–19% annually. Admission duration shortened by 2% annually (*p* < 0.0001). An intravenous thrombolysis was given in four admissions. It seems that migraine hospital admissions have become more frequent and the patients more often have cardiovascular risk factors, suggesting increased awareness and more aggressive acute evaluation of suspected stroke as the cause.

## 1. Introduction

Migraine affects almost 2 billion people worldwide, over 80 million people in Western Europe and approximately 1 million persons in Finland. It is among the most important causes of disability worldwide, especially in young adults and middle-aged women. [1]. Patients with migraine have an increased need of health care services, but recent data on the need of hospital care and characteristics of migraine related admissions are lacking [2,3,4,5,6]. Moreover, while awareness of stroke has increased and the hyperacute care of stroke has become much more active, migraine is recognized as one of the most common stroke mimics [7,8]. Considering that even up to 40% of acute stroke diagnoses are false positives [9], we suspect that the hospital admission rates for migraine may also have coincidentally increased. This could also be reflected in the admitted patients’ cardiovascular risk factor profile. Therefore, we performed a nationwide retrospective registry study on all migraine admissions over a decade in Finland.

## 2. Methods

### 2.1. Data Collection

All discharges from neurological, internal medicine, and pediatric wards on mainland Finland with migraine (ICD-10 code G43.X) as the primary diagnosis between 1 January 2004–31 December 2014 were identified from the Care Register for Health Care (CRHC), a mandatory database for all public health care hospital discharges in the country. Comorbidities were analyzed based on ICD-10 codes. Charlson comorbidity index (CCI) score including age was calculated as previously described [10]. Only patients at least 16 years of age were included. The study was approved by the National Institute for Health and Welfare of Finland (permissions no: THL/143/5.05.00/2015 and THL/1349/5.05.00/2015).

### 2.2. Statistical Analyses

Count variables were analyzed with Poisson regression modeling, in-hospital mortality was analyzed with Cox regression, and length of hospital stay (log transformed and standardized due to skewness) with linear regression. Statistical significance was inferred at (two-tailed) *p*-value < 0.05. All analyses were conducted using IBM SPSS Statistics for Windows, Version 24.0 (IBM Corp. Released 2016. Armonk, NY, USA: IBM Corp.).

## 3. Results

**Figure 1:**
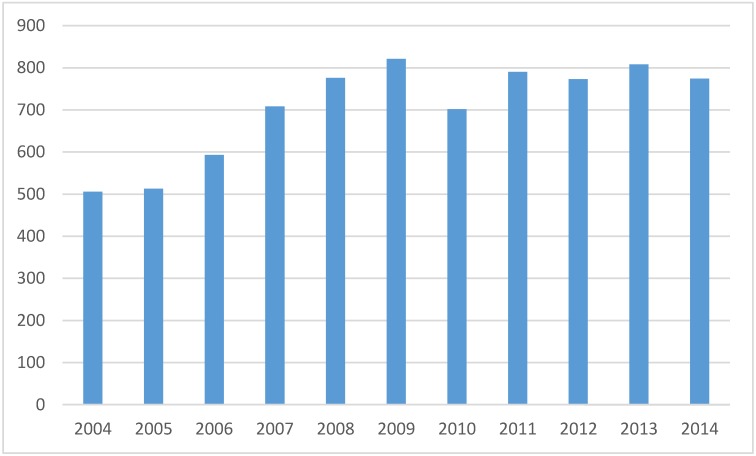
Frequency of migraine admissions by year in Finland.

We identified 6195 individuals with a total of 7764 migraine discharges (range 1–32 discharges/person, median 1). There were 175 patients with more than 2 discharges, 61 with 5 or more, and 18 with more than 10. The annual number of discharges was 506 in 2004 and 774 in 2014 (Figure 1), increasing annually by 4.4% (95% CI 3.0–5.8%, *p* < 0.0001) in women and 3.1% (95% CI 1.4–4.9, *p* = 0.00046) in men. Women (6354 discharges, median age 39.0 years, IQR 22) were younger than men (1410 discharges, median age 42.0, IQR 21, *p* < 0.001) with no change during the study period either in men (*p* = 0.055) or women (*p* = 0.29). Most of the discharges were from neurology wards (91.2%), with nearly all the rest from internal medicine wards (8.6%).

The CCI score was higher in men (0.28, SD 0.70) than women (0.22, SD 0.59, *p* = 0.003). Comorbidity burden had an increasing trend during the study period (estimated annual increase 10% in men and 12% in women, *p* < 0.0001 for both). Atrial fibrillation was recorded in 108 discharges (increasing 17% annually, *p* = 0.000009), diabetes in 138 (increasing 11% annually, *p* < 0.00028), hypercholesterolemia in 221 (increasing 19% annually, *p* < 0.000001), and hypertension in 564 discharges 0.00028 (increasing 11% annually, *p* < 0.000001).

Mean duration of hospital admission was 2.9 days (SD 2.2) for men and 2.8 days (SD 1.9) for women (*p* = 0.028). Duration of admissions shortened by an estimated 2% per year in both sexes (*p* < 0.0001 for both). No patient died while in hospital. An intravenous thrombolysis was given in four admissions (0.05%; ages 37–58, two women).

## 4. Discussion

This population-based, nationwide registry study showed that the number of hospital admissions due to migraine increased 4.2% annually from 2004 to 2014 in Finland. Moreover, the comorbidity burden of the admitted patients increased, and the prevalence of common stroke risk factors increased 11–19% annually, although the low CCI score suggests that the patients were rather healthy in general. In contrast, cardiovascular risk factor profiles have developed favorably in Finnish general population [11,12,13]

From 1990 to 2016, the age-standardized prevalence of migraine decreased by 1.2% in Western Europe and by 1.8% in Finland [1]. Therefore, either the need of hospital treatment per patient has markedly increased in Finland during the study period, or there are factors unrelated to migraine treatment driving the admission trend. Since there were no new developments in the treatment of acute migraine during the study period but the admission duration decreased, it seems that the latter was the case. There were also no significant changes in the clinical approach to migraine, as a headache disorder, in Finland during the study period.

The patients in this study appeared slightly older than average patients with status migraenosus admitted to a hospital [14]. Considering that the age of the admitted patients remained stable but their comorbidity burden, and particularly the frequency of stroke risk factors increased, we suggest that the trend may have been influenced by the known association between migraine and stroke. Not only is migraine one of the most common stroke mimics [7,8], it is also associated with stroke pathophysiology and occurrence in a way that has become better known and acknowledged during the recent decades [8,15]. Furthermore, clinical diagnoses are not always accurate [16]. Increased awareness and more aggressive acute treatment of stroke may therefore have resulted in more migraine admissions to rule out stroke by advanced imaging and/or clinical follow-up. The association of migraine and stroke here is also supported by the fact that four patients with migraine in our data received an intravenous thrombolysis, although this proportion seems low when compared to other studies [8].

Interestingly, there was no change in the annual stroke admission frequency of the working-aged in Finland in the same period [17], suggesting that an increasing number of patients were screened for no apparent benefit. At the same time, the number of hospital beds per 1000 population decreased from 7.1 to 4.5 [18], meaning that this increasingly scarce resource must be managed optimally. Interestingly, according to OECD data, there were 15.3 MRI scanners per million population in Finland in 2007, and in 2014 the figure was 23.3 per million. Increased availability of advanced imaging may in itself have influenced the admission trends as more accurate diagnostics have become feasible.

A major limitation of the current study was its retrospective nature, as relying on registry data has obvious weaknesses. Apart from comorbidities, we did not have data on patients’ employment or socioeconomic status, previously identified risk factors, medication, or details of clinical presentation. The major strength of our study was the availability of complete national data spanning a decade from a reliable registry [19]. As acute care requiring hospitalization in Finland is almost exclusively the domain of the public health care system, we believe that this nationwide study represents true population incidence of migraine hospitalizations and the changes therein.

In conclusion, the frequency of hospital admissions due to migraine has increased in Finland and the admitted patients more often have cardiovascular risk factors. Increased awareness of the association between migraine and stroke, as well as more aggressive acute evaluation of suspected strokes, are suggested as the cause.
